# Integrated multi-omics identified the novel intratumor microbiome-derived subtypes and signature to predict the outcome, tumor microenvironment heterogeneity, and immunotherapy response for pancreatic cancer patients

**DOI:** 10.3389/fphar.2023.1244752

**Published:** 2023-09-07

**Authors:** Biao Zhang, Jifeng Liu, Han Li, Bingqian Huang, Bolin Zhang, Binyu Song, Chongchan Bao, Yunfei Liu, Zhizhou Wang

**Affiliations:** ^1^ Department of General Surgery, The First Affiliated Hospital of Dalian Medical University, Dalian, China; ^2^ Department of Oncology, Southwest Medical University, Luzhou, China; ^3^ Institute (College) of Integrative Medicine, Dalian Medical University, Dalian, China; ^4^ Department of Visceral, Martin-Luther-University Halle-Wittenberg, University Medical Center Halle, Halle, Germany; ^5^ Department of Plastic Surgery, Xijing Hospital, Xi’an, China; ^6^ Department of Breast and Thyroid Surgery, Affiliated Hospital of Youjiang Medical University for Nationalities, Baise, China; ^7^ Department of General, Visceral, and Transplant Surgery, Ludwig-Maximilians-University Munich, Munich, Germany

**Keywords:** microbiome, pancreatic cancer, prognosis, tumour microenvironment, immunotherapy, single-cell analysis

## Abstract

**Background:** The extremely malignant tumour known as pancreatic cancer (PC) lacks efficient prognostic markers and treatment strategies. The microbiome is crucial to how cancer develops and responds to treatment. Our study was conducted in order to better understand how PC patients’ microbiomes influence their outcome, tumour microenvironment, and responsiveness to immunotherapy.

**Methods:** We integrated transcriptome and microbiome data of PC and used univariable Cox regression and Kaplan–Meier method for screening the prognostic microbes. Then intratumor microbiome-derived subtypes were identified using consensus clustering. We utilized LASSO and Cox regression to build the microbe-related model for predicting the prognosis of PC, and utilized eight algorithms to assess the immune microenvironment feature. The OncoPredict package was utilized to predict drug treatment response. We utilized qRT-PCR to verify gene expression and single-cell analysis to reveal the composition of PC tumour microenvironment.

**Results:** We obtained a total of 26 prognostic genera in PC. And PC samples were divided into two microbiome-related subtypes: Mcluster A and B. Compared with Mcluster A, patients in Mcluster B had a worse prognosis and higher TNM stage and pathological grade. Immune analysis revealed that neutrophils, regulatory T cell, CD8^+^ T cell, macrophages M1 and M2, cancer associated fibroblasts, myeloid dendritic cell, and activated mast cell had remarkably higher infiltrated levels within the tumour microenvironment of Mcluster B. Patients in Mcluster A were more likely to benefit from CTLA-4 blockers and were highly sensitive to 5-fluorouracil, cisplatin, gemcitabine, irinotecan, oxaliplatin, and epirubicin. Moreover, we built a microbe-derived model to assess the outcome. The ROC curves showed that the microbe-related model has good predictive performance. The expression of LAMA3 and LIPH was markedly increased within pancreatic tumour tissues and was linked to advanced stage and poor prognosis. Single-cell analysis indicated that besides cancer cells, the tumour microenvironment of PC was also rich in monocytes/macrophages, endothelial cells, and fibroblasts. LIPH and LAMA3 exhibited relatively higher expression in cancer cells and neutrophils.

**Conclusion:** The intratumor microbiome-derived subtypes and signature in PC were first established, and our study provided novel perspectives on PC prognostic indicators and treatment options.

## 1 Introduction

An extremely dangerous tumour of the digestive tract, pancreatic cancer (PC) has a sneaky onset and quick progression. Clinical practise for PC lacks efficient therapeutic medications, and the prognosis is extremely poor (Chi et al., 2022a; Zhang B. et al., 2023). With over 459,000 new cases and 432,000 fatalities per year, epidemiological studies have shown that PC is the seventh greatest cause of cancer-related deaths globally (Ryan et al., 2014; Bray et al., 2018). Since most PCs are not discovered until they are advanced, the probability of surviving more than 5 years is low, at only 9% (Mizrahi et al., 2020; Siegel et al., 2020). Surgery combined with adjuvant chemotherapy is currently the standard treatment for PC. However, due to the complexity and heterogeneity of the tumour microenvironment of PC, it often leads to the generation of treatment resistance and the differential response of different patients to treatment. Therefore, the development of indicators for early detection as well as risk assessment is an important clinical problem to be solved urgently. So far, many studies have developed biomarkers for early diagnosis and risk assessment of PC from the perspectives of subcellular organelle function, tumour immune response, and gene modification (Romero et al., 2020; Xiao et al., 2022; Zhuo et al., 2022). Nevertheless, clinically effective early diagnostic markers, therapeutic targets, and risk assessment schemes in PC are still lacking.

Recent studies have shown that microbes have been considered to influence the occurrence, development, metastasis, as well as therapy response of different tumours, especially closely related to tumour microenvironment and immune response (Sepich-Poore et al., 2021). Many basic researches revealed that intratumor microbiome could affect the progression, metastases, prognosis, as well as immunotherapy of cancer patients by regulating oxidative stress, Toll-like receptors-mediated immune response, and tumour cell metabolism, involving mTOR, STAT3, Wnt, MAPK and other signaling pathways (Pushalkar et al., 2018; Wang et al., 2019; Wei et al., 2019). The diversity and composition of microbiome play crucial functions in the prognosis of PC, and can regulate the tumour immune microenvironment (Riquelme et al., 2019; Kartal et al., 2022). Mao et al. (Mao et al., 2022) have constructed the intratumor microbiome signature for breast cancer to predict the outcome. However, the correlation of intratumor microbiome with the clinicopathological features, prognosis, tumour microenvironment heterogeneity, and therapeutic response in PC is still not reported.

Our study first constructed intratumor microbiome-derived subtypes for PC by integrating microbiome and transcriptome data, and comprehensively analyzed the important role of microbiome in clinicopathological characteristics, prognosis, tumour immune microenvironment, and immunotherapy response of PC patients. Meanwhile, we also identified the microbiome-related differentially expressed genes and utilized them to build a prognostic model. Subsequently, we verified the LIPH and LAMA3 mRNA expression by real-time quantitative PCR (qRT-PCR). Finally, we used single-cell analysis to further reveal the cell subpopulation composition in pancreatic tumour microenvironment, as well as the relative expression of LIPH and LAMA3 in different cell subpopulations. This study can provide innovative ideas for the outcome assessment as well as therapy of PC.

## 2 Materials and methods

### 2.1 Data acquisition

Transcriptome data (containing 178 PC tissues and 4 paracancerous tissues) of PC, along with clinical data (containing 185 PC samples), were downloaded via The Cancer Genome Atlas (TCGA, https://portal.gdc.cancer.gov/). Microbiome data of PC were obtained via the cBioPortal platform (https://www.cbioportal.org/) (Cerami et al., 2012; Mao et al., 2022). Gene expression data and survival information for GSE62452 (containing 69 PC tissues), GSE28735 (containing 45 PC tissues and paracancerous tissues), and GSE57495 (containing 69 PC tissues and paracancerous tissues) datasets were obtained via the Gene Expression Omnibus database (GEO, https://www.ncbi.nlm.nih.gov/geo/). Using “sva” R package to eliminate batch effects between different datasets (Chi et al., 2023a). Microbiomes associated with PC prognosis were identified by univariable Cox regression analysis as well as Kaplan-Meier (KM) method (Chi et al., 2023b). These microbes associated with PC prognosis were used in subsequent analyses.

### 2.2 Clustering analysis

Consensus cluster was carried out utilizing “ConsensusClusterPlus” R package for PC samples based on the relative abundance of prognosis-related microbes (Zhang X. et al., 2023). The ideal clustering number was established based on the cumulative distribution function (CDF) curve as well as variations of CDF curve area. Using principal component analysis (PCA) as well as t-distributed stochastic neighbor embedding (t-SNE) analyses to demonstrate the accuracy of our clustering findings. Then the prognosis and clinical pathological features between different subtypes were further compared. Additionally, the differences in the relative abundance of prognosis-associated microbes among different subtypes were compared.

### 2.3 Gene set enrichment analysis (GSEA)

Gene set enrichment analysis (GSEA) was performed using the gene sets “c2.cp.kegg.v7.5.1.symbols.gmt” as well as “c5.go.v7.5.1.symbols.gmt” to compare the putative mechanisms behind the variations between the different intratumor microbiome subtypes (Subramanian et al., 2005). GSEA enrichment analysis was conducted using the R packages “limma”, “org.Hs.e.g.,.db”, “clusterProfiler”, and “enrichplot”. An adjusted *p*-value <0.05 were considered statistically significant.

### 2.4 Immune analysis

To analyze the differences in tumour immune microenvironment among different intratumor microbiome subtypes, the stromal, immune, and ESTIMATE score of every PC patient were evaluated utilizing “ESTIMATE” algorithm (Yoshihara et al., 2013). For evaluating the variations in infiltrated levels of immune cell subset between different subtypes, the infiltration scores calculated utilizing eight algorithms were downloaded from the Tumor Immune Estimation Resource database (TIMER, http://timer.cistrome.org/) (Yuan et al., 2022). The Cancer Immunome Atlas (TCIA, https://tcia.at/) is a database developed based on the TCGA database, which analyzes the tumour immune microenvironment and tumor antigen genes in 20 solid tumours (Charoentong et al., 2017). The immunophenoscore (IPS) of PC patients from TCIA database were downloaded. Then the differences in the responsiveness to cytotoxic T lymphocyte antigen-4 (CTLA-4) and programmed cell death protein 1 (PD-1) blockers between the different intratumor microbiome subtypes were further analyzed.

### 2.5 Drug sensitivity analysis

OncoPredict is an R package created via Maeser et al., which is used to predict drug response and biomarkers *in vivo* or in cancer patients based on cell line screening data (Maeser et al., 2021). OncoPredict was employed to assess the variations of drug sensitivity between the patients with different intratumor microbiome subtypes.

### 2.6 Differential expression analysis

In order to further analyze the differences between intratumor microbiome subtypes, we utilized “limma” package to find differentially expressed genes (DEGs), which were defined as intratumor microbe-related DEGs (Chi et al., 2022b). The filtering criteria were |log_2_FC| > 1, the adjusted *p*-value <0.05. Additionally, PC patients of TCGA, GSE28735, GSE62452, and GSE57495 datasets were merged for identifying DEGs between PC tissues and paracancerous tissues. The DEGs obtained from both approaches were then combined, then we utilized Gene Ontology (GO) and Kyoto Encyclopedia of Genes and Genomes (KEGG) enrichment analyses to assess the biological processes and functions they participate in (Ashburner et al., 2000; Kanehisa and Goto, 2000). For assessing the prognostic value of these DEGs in PC, consensus clustering was carried out. Survival times of the various subtypes were compared using KM curve.

### 2.7 Construction of the prognostic signature

For accurately assessing the prognosis of individual PC samples, we employed LASSO regression and Cox regression analysis to build a prognostic signature utilizing intratumor microbe-related DEGs. Samples from TCGA dataset were grouped into a training cohort as well as an internal validation cohort utilizing “caret” package in a 5:5 ratio, while samples from GEO dataset were utilized as an external validation cohort. The score for every sample could be computed using risk score formula. Additionally, a comparison with the median score of the training cohort was used to categorise each sample into high- or low-risk score categories or groups. Utilizing KM curve to compare the prognosis between different risk categories. The performance of the signature was appraised by plotting time-dependent receiver operating characteristic (ROC) curve as well as figuring out the area under the curve (AUC).

### 2.8 Correlation of clinicopathological features, independent prognostic analysis, and construction of nomogram prediction model

Our study combined the clinicopathological information of PC samples with the risk scores and grouped them based on clinicopathological characteristics. Risk scores between different categories were compared utilizing Wilcoxon signed-rank test and Kruskal–Wallis rank sum test. Utilizing univariable as well as multivariable Cox regression to identify the independent prognostic factor of PC. Subsequently, the clinicopathological features and risk score were utilized for building a nomogram prediction model by “rms” R package (Park, 2018). Utilizing calibration curve to appraise the predictive accuracy of the nomogram.

### 2.9 Expression analysis of model genes and single-cell analysis

The GEPIA platform (http://gepia.cancer-pku.cn/) can allow for gene differential expression as well as survival analysis utilizing data of TCGA and GTEx databases (Tang et al., 2017). We utilized it to examine the expressed variations in LAMA3 and LIPH at RNA level between pancreatic cancer and normal tissues as well as the prognostic significance of LAMA3 and LIPH. Human Protein Atlas database (HPA, version22, https://www.proteinatlas.org/) aims at creating expressed patterns in protein of cells as well as tissues (Pontén et al., 2011). We can download immunohistochemistry images of pancreatic cancer and normal tissues via HPA platform. The tumor immune single-cell hub database (TISCH, http://tisch.comp-genomics.org) as a single-cell RNA-seq platform, focuses on the tumour microenvironment. It was utilized to reveal the composition of various cell subpopulations in the pancreatic tumour microenvironment and the relative expressed level of LAMA3 and LIPH within various cell subpopulations (Sun et al., 2021).

### 2.10 Real-time quantitative PCR

RNAs were extracted from cell lines, including a normal pancreatic epithelial cell line (HPDE6-C7) as well as three PC cell lines (CF-PAC1, PANC-1, and BxPC-3). The cDNAs were prepared using Reverse Transcription Reagent. Subsequently, PCR was performed. GAPDH served as the reference standard. Utilizing the ΔΔCt method to illustrate the relative expressed level of LAMA3 and LIPH. The primer sequences for human genes, including LAMA3 (Forward: 5′-ATT​GAA​TTG​AGC​ACC​AGC​GAT​AGC-3′, Reverse: 5′-CGA​TGA​GAA​GCC​GTA​GTC​CAG​AG-3′) as well as LIPH (Forward: 5′-TAC​GGG​ACT​AAA​TGT​GAG​GC-3′, Reverse: 5′-CCT​AGA​CTT​ACT​CCG​ATC​ATG-3′).

### 2.11 Data analysis

Data analysis was performed utilizing R (Version 4.1.2) as well as GraphPad Prism 9. For normally distributed quantitative data, utilizing *t*-test to compare the differences. For non-normally distributed quantitative data, utilizing Wilcoxon signed-rank test to compare the differences between two groups, and utilizing Kruskal–Wallis rank sum test to compare the differences among multiple groups. KM curve was utilized to compare the prognosis between different subtypes or categories. The *p*-value <0.05 represented remarkable significance.

## 3 Results

### 3.1 Identification of intratumor microbiome-derived subtypes

**FIGURE 1:**
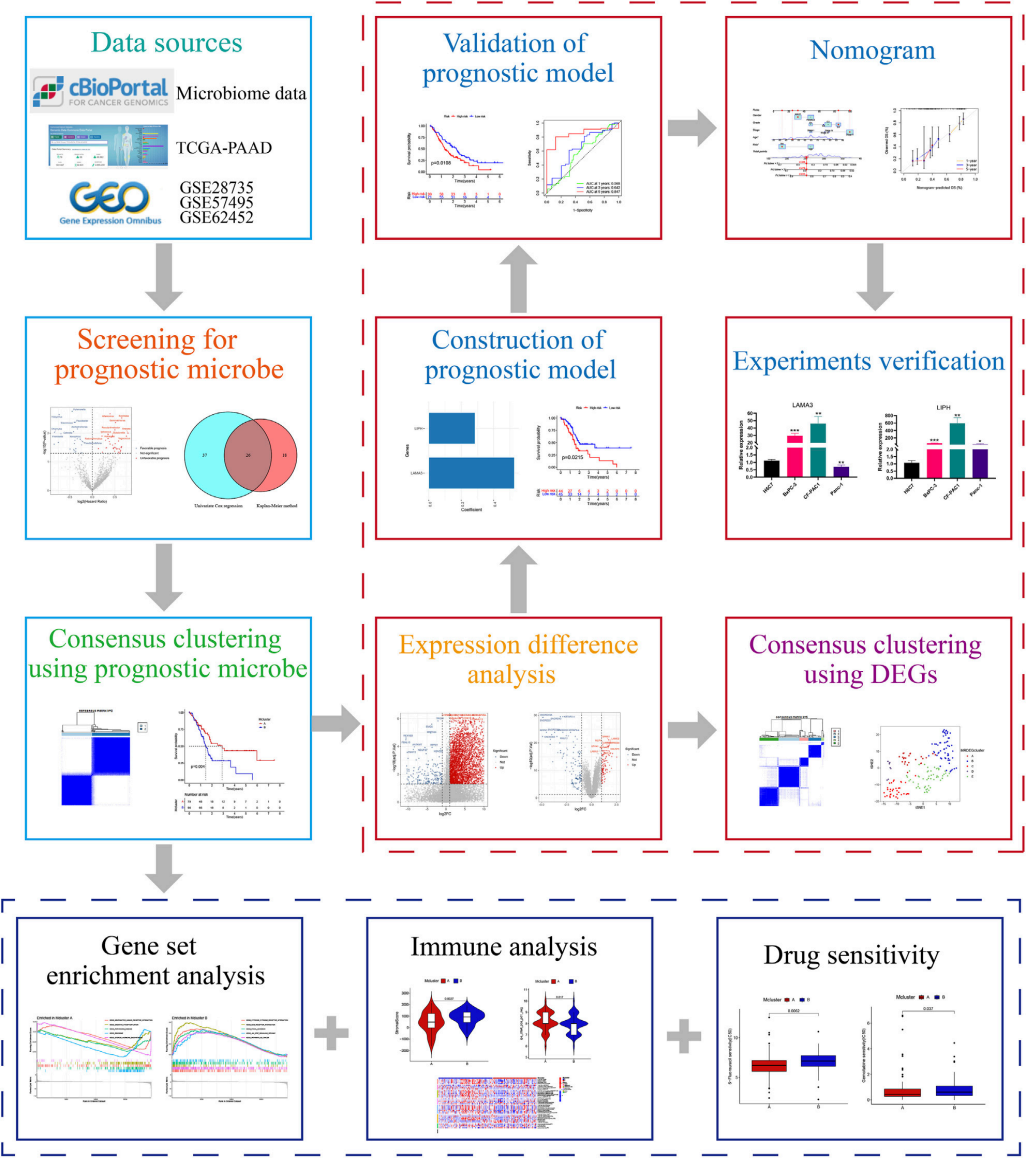
The study’s general procedure.

**FIGURE 2:**
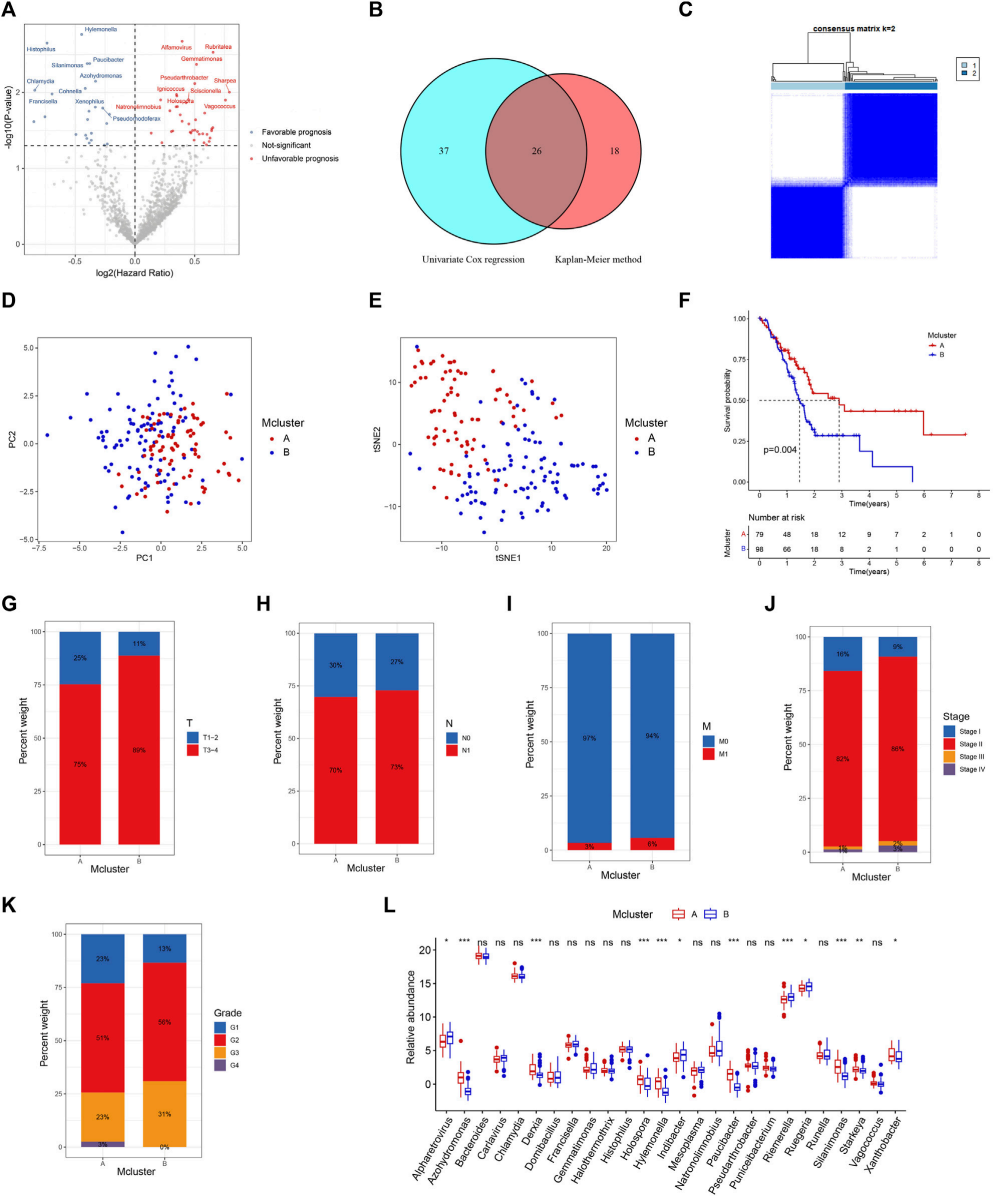
Identification of intratumor microbiome-derived subtypes. **(A)** Volcanic map of prognostic genera screened by univariable Cox regression. **(B)** Intersection of prognostic genera found by univariable Cox regression and Kaplan-Meier method. **(C)** Heatmap of consensus matrix when the cluster number was 2. PCA **(D)** and t-SNE **(E)** can clearly distinguish samples between Mcluster A and B. **(F)** Survival curves for Mcluster A and B. The proportion of T **(G)**, N **(H)**, M **(I)**, TNM **(J)** stage and pathological grade **(K)** between Mcluster A and B. **(L)** Differences in genera abundance between Mcluster A and B. (ns, no significant; *p < 0.05; **p < 0.01; ***p < 0.001).

The workflow of our research was depicted in Figure 1. Totally 1406 genera were obtained from the pancreatic tumour microenvironment through the cBioPortal platform (Supplementary Table S1). Univariate Cox regression analysis identified 63 genera associated with the prognosis of PC, with 24 genera associated with a favorable prognosis and 39 genera associated with a poor prognosis (Figure 2A). KM method identified 44 genera related to PC patients’ prognosis (Supplementary Table S2). The intersection of genera obtained from univariate Cox regression analysis and Kaplan-Meier analysis yielded 26 genera: Alpharetrovirus, Azohydromonas, *Bacteroides*, Carlavirus, *Chlamydia*, Derxia, Domibacillus, Francisella, Gemmatimonas, Halothermothrix, Histophilus, Holospora, Hylemonella, Indibacter, Mesoplasma, Natronolimnobius, Paucibacter, Pseudarthrobacter, Puniceibacterium, Riemerella, Ruegeria, Runella, Silanimonas, Starkeya, Vagococcus, and Xanthobacter (Figure 2B). Correlation analysis revealed complicated relationships among the 26 genera. For example, Vagococcus had positive correlations with Puniceibacterium, Halothermothrix, Derxia, Starkeya, Pseudarthrobacter, Domibacillus, Gemmatimonas, and Silanimonas, while had negative correlations with Ruegeria, *Chlamydia*, Francisella, Carlavirus, and Alpharetrovirus (Supplementary Figure S1). Subsequently, consensus clustering was carried out utilizing the abundance of 26 genera. The CDF curve as well as the area variation under the curve were depicted in Supplementary Figures S2, S3, which indicated the ideal k value was 2. The consensus matrix at *k* = 2 was shown in Figure 2C. PC patients were divided into two intratumor microbiome-derived subtypes: Mcluster A and Mcluster B. PCA as well as t-SNE can clearly differentiate samples of Mcluster A and Mcluster B (Figures 2D, E). Survival analysis indicated a significantly better prognosis for Mcluster A compared to Mcluster B (Figure 2F). Compared to Mcluster B, a higher proportion of T1-2 stage, N0 stage, M0 stage, Stage I, and pathological grade G1 was observed in Mcluster A (Figures 2G–K). Furthermore, the abundance differences of 26 genera in different subtypes were analyzed. The results showed that Azohydromonas, Derxia, Holospora, Hylemonella, Paucibacter, Silanimonas, Starkeya, and Xanthobacter had remarkably higher abundance in Mcluster A, while Alpharetrovirus, Indibacter, Riemerella, and Ruegeria had remarkably higher abundance in Mcluster B (Figure 2L).

### 3.2 Gene set enrichment analysis

**FIGURE 3:**
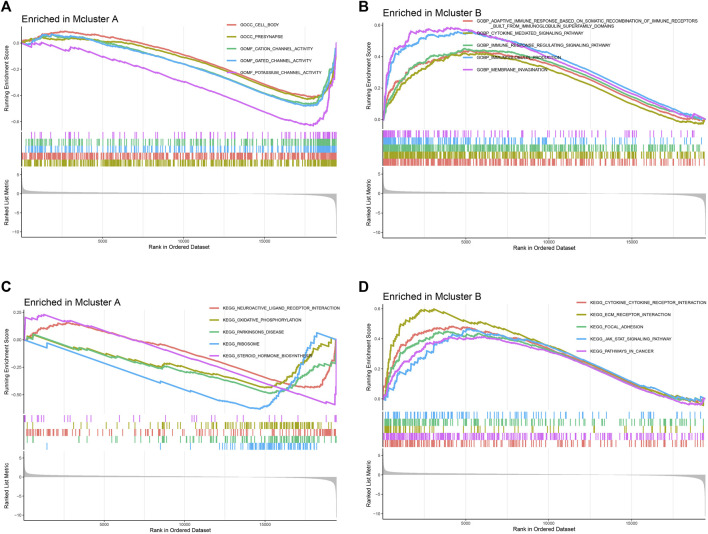
Gene set enrichment analysis. The remarkably enriched pathways of Mcluster A **(A)** and B **(B)** in “c5.go.v7.5.1.symbols.gmt” gene set. The remarkably enriched pathways of Mcluster A **(C)** and B **(D)** in “c2.cp.kegg.v7.5.1.symbols.gmt” gene set.

For investigating putative molecular mechanisms between different intratumor microbiome-derived subtypes, we performed GSEA analysis. In “c5.go.v7.5.1.symbols.gmt” gene set, we found that Mcluster A was mainly enriched in cell body, presynapse, gated channel activity, as well as potassium channel activity (Figure 3A). And Mcluster B was significantly more abundant in cytokine-mediated signaling pathway, immune response-regulating signaling pathway, immunoglobulin production, as well as membrane invagination (Figure 3B). In “c2.cp.kegg.v7.5.1.symbols.gmt” gene set, Mcluster A was found to be enriched in pathways containing oxidative phosphorylation, Parkinson’s disease, ribosome, and steroid hormone biosynthesis (Figure 3C). Conversely, Mcluster B was enriched in pathways including cytokine-cytokine receptor interaction, ECM-receptor interaction, focal adhesion, JAK-STAT signaling pathway, etc (Figure 3D).

### 3.3 Immune analysis

**FIGURE 4:**
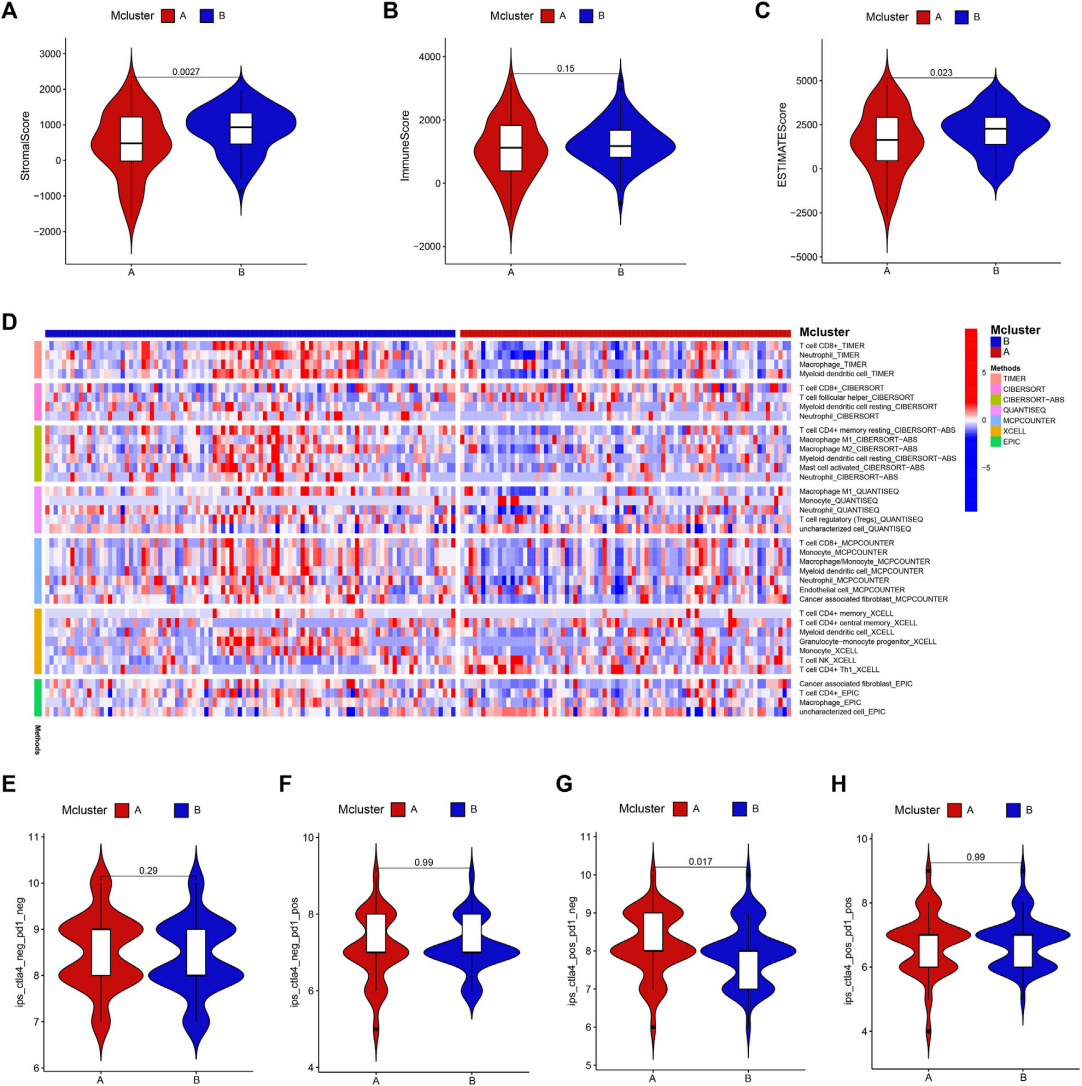
Immunoassay. The stroma **(A)**, immune **(B)** as well as ESTIMATE **(C)** scores between Mcluster A and B were evaluated using “ESTIMATE” algorithm. **(D)** Variations in infiltrated levels of different immune cell subpopulations in Mcluster A and B. The overall immunophenoscore **(E)**, immunophenoscore for PD-1 blocker **(F)**, CTLA-4 blocker **(G)**, and immunophenoscore for CTLA-4 and PD-1 blocker **(H)** between Mcluster A and B.

We evaluated the variations in immune infiltration between distinct subtypes using various algorithms to investigate the association between intratumor microbiome-derived subtypes and tumour microenvironment. The “ESTIMATE” algorithm showed that Mcluster B had higher stroma as well as ESTIMATE score (Figures 4A, C), whereas there was no discernible difference in immunological score between Mcluster A and Mcluster B (Figure 4B). For investigating the infiltrated variations in immune cell subpopulations between different subtypes, the infiltrated scores of immune cell subpopulations were obtained from the TIMER database. We found that neutrophils, regulatory T cell (Treg), CD8^+^ T cell, macrophages M1 and M2, cancer-associated fibroblasts, myeloid dendritic cell, as well as activated mast cell exhibited remarkably higher infiltration levels within Mcluster B (Figure 4D). Then, we further utilized the TCIA platform to investigate the response of PC to immune checkpoint blockers. In the matter of overall immunophenoscore, immunophenoscore for PD-1 blocker, and immunophenoscore for CTLA-4 and PD-1 blocker, our investigation could not detect any remarkable variations between the two subtypes (Figures 4E, F, H). However, the immunophenoscore for CTLA-4 blocker had a remarkably higher score in Mcluster A (Figure 4G), suggesting that patients with PC in Mcluster A may have a better response to CTLA-4 blockers.

### 3.4 Drug sensitivity analysis

**FIGURE 5:**
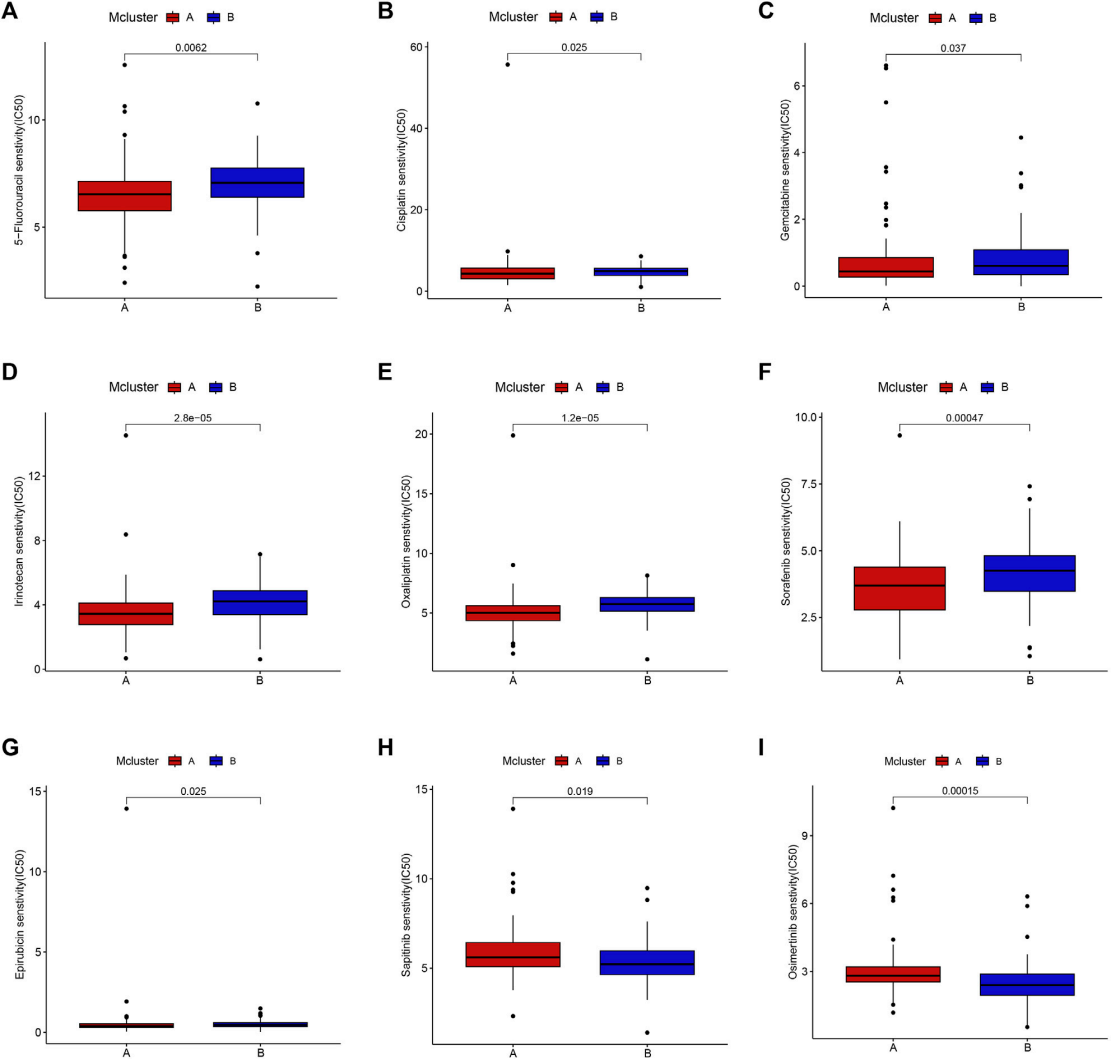
Drug sensitivity between Mcluster A and B. Patients with Mcluster A had high sensitivity to 5-Fluorouracil **(A)**, Cisplatin **(B)**, Gemcitabine **(C)**, Irinotecan **(D)**, Oxaliplatin **(E)**, Sorafenib **(F)**, and Epirubicin **(G)**. Patients with Mcluster B were more sensitive to Sapitinib **(H)** and Osimertinib **(I)**.

Drug adjuvant therapy is an important means for enhancing PC patients’ prognoses. Nevertheless, the emergence of primary and secondary drug resistance often leads to treatment failure. To enhance the curative impact, it is crucial to choose medications with high sensitivity for various patients. Our results showed that patients in Mcluster A were more sensitive to 5-fluorouracil, cisplatin, gemcitabine, irinotecan, oxaliplatin, sorafenib, and epirubicin (Figures 5A–G), while patients in Mcluster B were more sensitive to sapitinib and osimertinib (Figures 5H, I). Thus, intratumor microbiome-derived subtypes can provide new strategies for personalized therapy in PC.

### 3.5 Identification and analysis of differentially expressed genes

**FIGURE 6:**
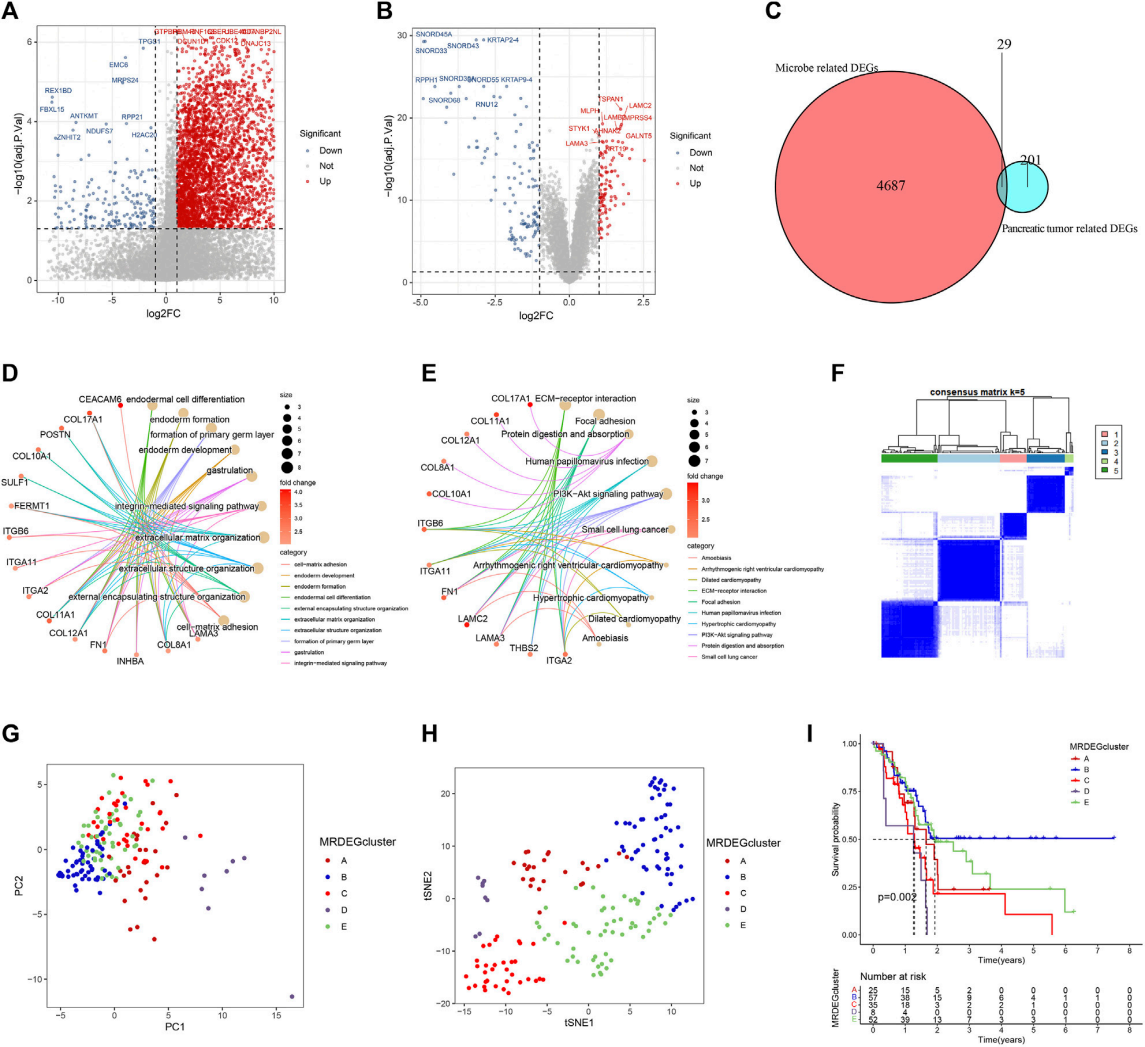
Identification and analysis of differentially expressed genes (DEGs). **(A)** Volcanic map of DEGs between Mcluster A and B. **(B)** Volcanic map of DEGs between PC tissues and normal tissues. **(C)** Intersection of DEGs. **(D)** GO enrichment analysis. **(E)** KEGG enrichment analysis. **(F)** Heatmap of consensus matrix when the cluster number was 5. PCA **(G)** and t-SNE **(H)** can clearly distinguish samples among different MRDEGclusters. **(I)** Survival curves among different MRDEGclusters.

To explore the transcriptional differences in genes among different intratumor microbiome-derived subtypes, we identified 4716 DEGs, with 281 genes having higher expression within Mcluster A as well as 4435 genes having higher expression within Mcluster B (Figure 6A). Additionally, between pancreatic tumour tissue and paracancerous tissues, we identified 230 DEGs, with 107 genes had higher expression within tumour tissues and 123 genes had higher expression within paracancerous tissues (Figure 6B). Further intersection analysis obtained 29 DEGs (Figure 6C). To analyze the biological processes associated with these 29 genes, we carried out GO as well as KEGG analyses. GO analysis identified the enriched pathways such as cell-matrix adhesion, endoderm development, endoderm formation, endodermal cell differentiation, extracellular matrix organization, formation of the primary germ layer, gastrulation, and integrin-mediated signaling pathway (Figure 6D). KEGG analysis revealed the enriched pathways containing amoebiasis, dilated cardiomyopathy, ECM-receptor interaction, focal adhesion, human papillomavirus infection, hypertrophic cardiomyopathy, PI3K-Akt signaling pathway, and small cell lung cancer (Figure 6E).

We further carried out consensus clustering analysis to investigate the prognostic usefulness of these 29 microbiome-related genes in PC. Five subtypes were identified for all PC patients: MRDEGclusters A through E (Figure 6F). PCA as well as t-SNE analysis clearly distinguished the five subtypes (Figures 6G, H). KM curves indicated that the five subtypes’ prognoses varied significantly, with MRDEGcluster B having the best prognosis and MRDEGcluster D having the worst prognosis (Figure 6I).

### 3.6 Building and testing a prognostic signature

**FIGURE 7:**
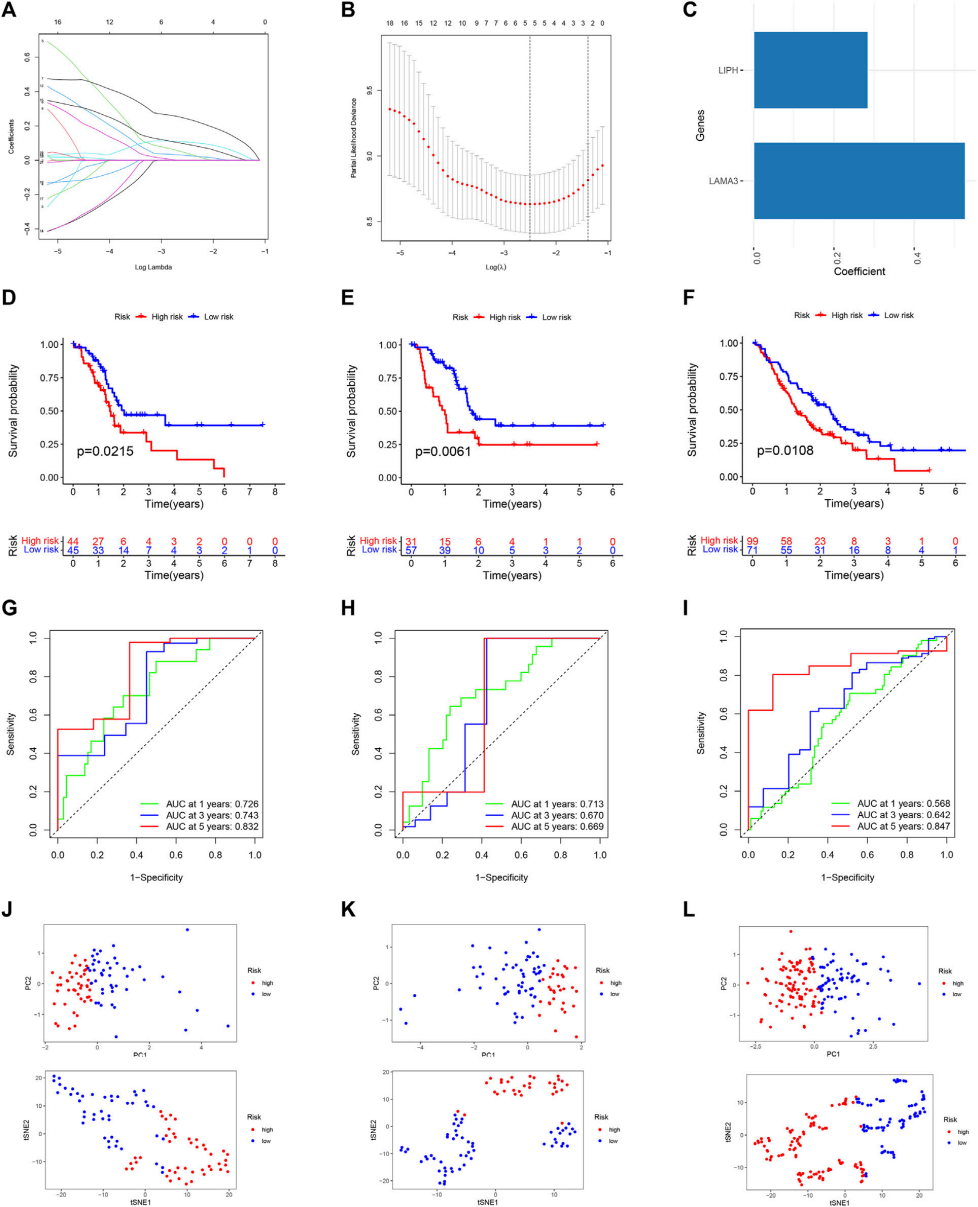
Building and testing a prognostic signature. **(A)** The coefficient path graph. **(B)** The cross validation curves. **(C)** Coefficient of LAMA3 and LIPH. Survival curve of the training **(D)**, internal validation **(E)**, and external GEO **(F)** dataset. The time-dependent ROC curve of the training **(G)**, internal validation **(H)**, and external GEO **(I)** dataset. PCA and t-SNE for the training **(J)**, internal validation **(K)**, and external GEO **(L)** dataset.

For predicting each PC patient’s prognosis more accurately, we constructed a prognostic signature utilizing microbiome-derived DEGs. Firstly, 21 genes linked to PC patients’ prognosis were found using univariable Cox regression analysis Supplementary Figure S4. Then, utilizing LASSO regression to eliminate genes overfitting (Figures 7A, B). Finally, utilizing multivariable Cox regression to construct the prognostic model, which included two genes: LIPH and LAMA3 (Figure 7C). PC patients with low-risk scores had considerably longer overall survival times than those with high-risk scores (Figure 7D). The prognostic model’s dependability was further attested to by the internal as well as external validation sets (Figures 7E, F). The AUC values of 1, 3, and 5-year survival rates were 0.726, 0.743, and 0.832 in the training set (Figure 7G), 0.713, 0.670, and 0.669 in the internal validation set **(** Figure 7H), and 0.568, 0.642, and 0.847 in the external validation set (Figure 7I), indicating good predictive value. PCA and t-SNE analyses clearly distinguished patients between high- and low-risk categories in the training (Figure 7J), internal validation (Figure 7K), and external validation sets (Figure 7L).

**FIGURE 8:**
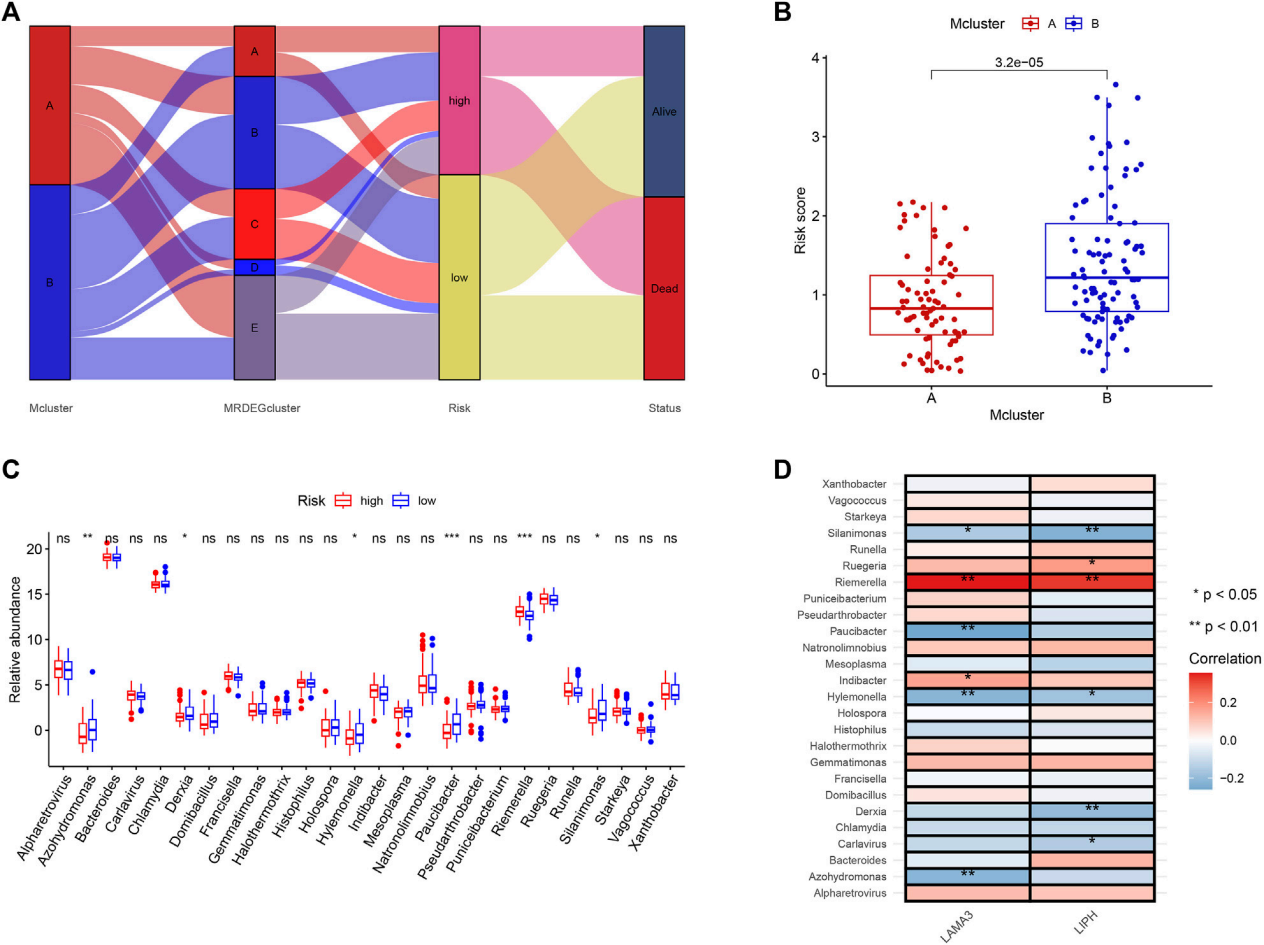
The correlation of microbiome-derived subtypes and signature. **(A)** Alluvial diagram for the microbiome-derived subtypes and signature. **(B)** Differences in risk score of Mcluster A and Mcluster B. **(C)** Variations in genera abundance between the high- and low-risk group. **(D)** Correlation of LAMA3 and LIPH and different genera. (ns, no significant; *p < 0.05; **p < 0.01; ***p < 0.001).

We used a Sankey diagram to illustrate the association between the prognostic model and intratumor microbiome-derived subtypes (Figure 8A). PC patients in Mcluster A had considerably lower risk scores than those in Mcluster B (Figure 8B). Besides, it was found that Riemerella had remarkably higher abundance in patients with high scores, while Azohydromonas, Derxia, Hylemonella, Paucibacter, and Silanimonas had remarkably higher abundance in patients with low scores (Figure 8C). Subsequently, we further investigated the association between gene expression and microbial abundance. Results showed that the abundance of Riemerella was remarkably related positively to the expression of LIPH and LAMA3, while the abundance of Silanimonas and Hylemonella was remarkably related negatively to the expression of LIPH and LAMA3 (Figure 8D).

### 3.7 Correlation of clinicopathological features, independent prognostic analysis, and construction of nomogram prediction model

**FIGURE 9:**
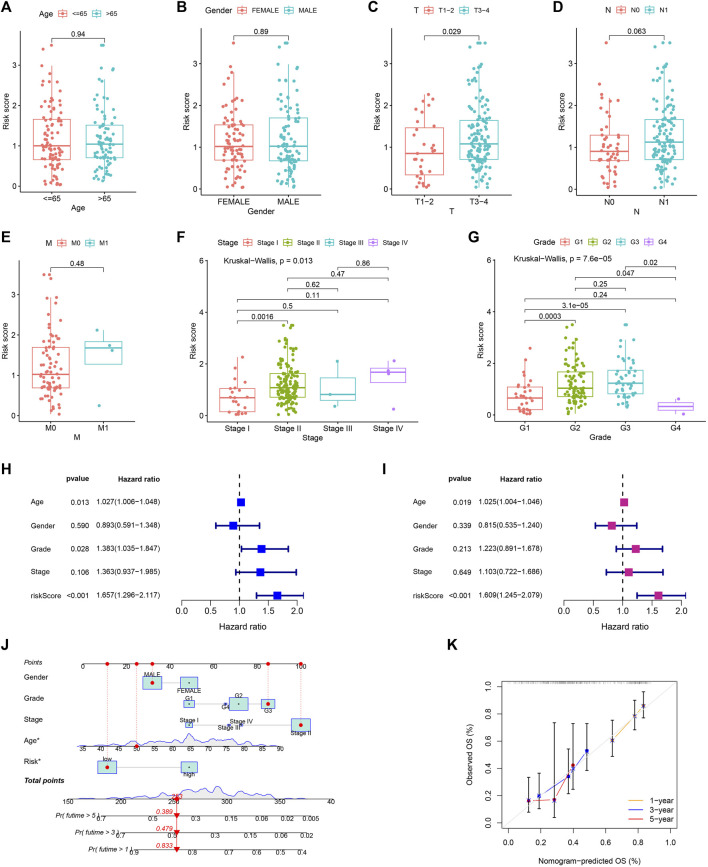
Independent prognostic analysis and constructing nomogram prediction model. The comparison of risk score in different age **(A)**, gender **(B)**, T **(C)**, N **(D)**, M **(E)**, TNM **(F)** stage, and pathological grade **(G)**. Forest map for univariate **(H)** and multivariate **(I)** Cox regression. **(J)** Nomogram prediction model. **(K)** Calibration curve of nomogram model.

Risk scores did not differ remarkably between groups generated by age, gender, and M stage (Figures 9A, B, E). Patients with N1 stage had higher risk scores compared to those with N0 stage, which were approaching statistical significance (Figure 9D). Patients’ risk scores were noticeably greater in those with higher T stage, TNM stage, and pathological grade (Figures 9C, F, G). Additionally, age and risk score were found to be independent poor prognostic variables of PC in both the univariable and multivariable Cox regression analyses (Figures 9H, I). Then, for further evaluating the prognosis of PC patients, we created a nomogram prediction model utilizing clinicopathological variables and risk scores (Figure 9J). The calibration curve demonstrated that our nomogram model had strong predictive value because the predictive 1, 3, and 5-year survival rates were relatively close to the actual 1, 3, and 5-year survival rates (Figure 9K).

### 3.8 Drug sensitivity analysis in different risk groups

**FIGURE 10:**
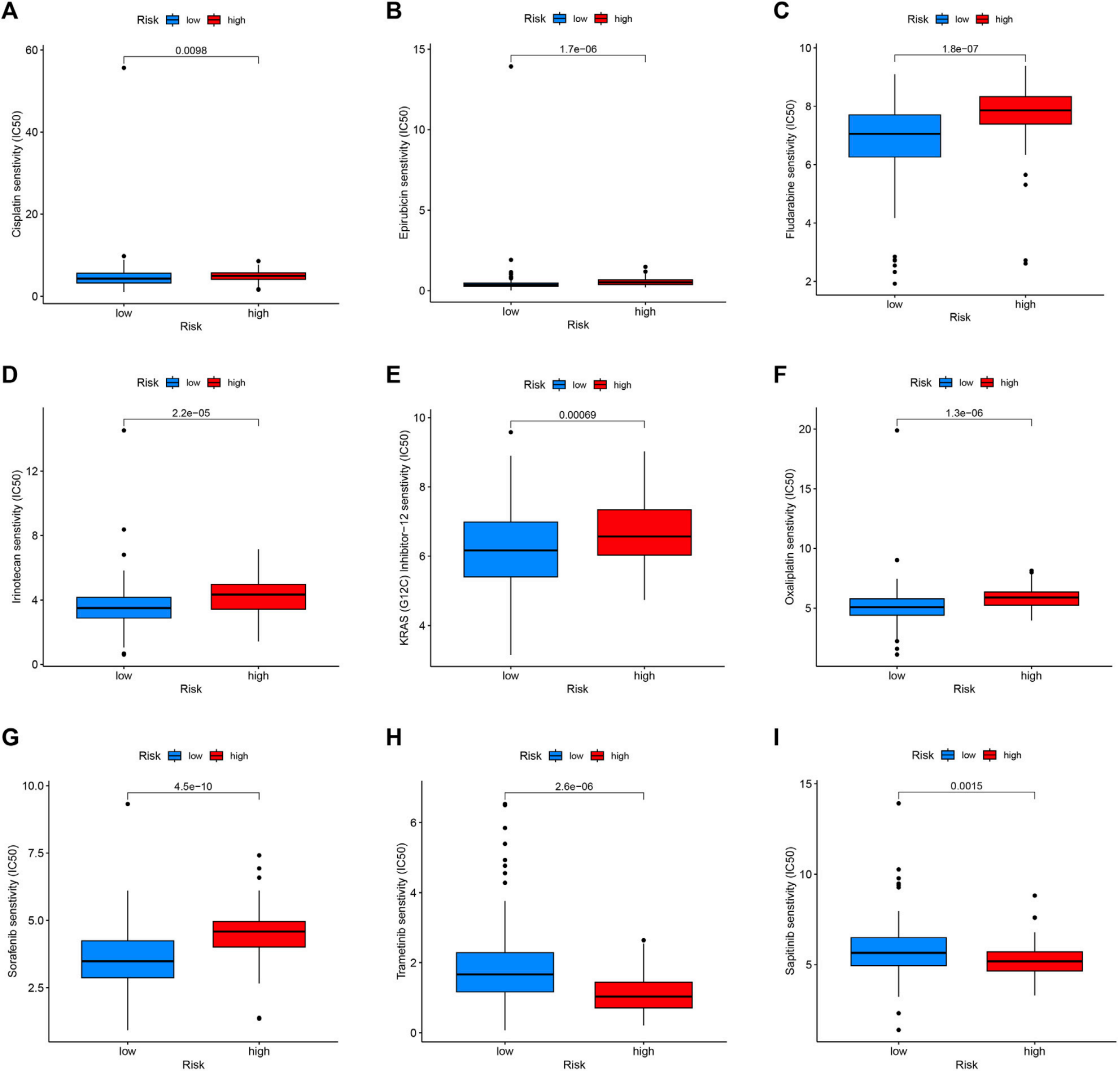
Drug sensitivity in the high- and low-risk group. Patients with low-risk scores were more sensitive to Cisplatin **(A)**, Epirubicin **(B)**, Fludarabine **(C)**, Irinotecan **(D)**, KRAS (G12C) Inhibitor-12 **(E)**, Oxaliplatin **(F)**, and Sorafenib **(G)**. Patients with high-risk scores had higher sensitivity to Trametinib **(H)** and Sapitinib **(I)**.

For exploring the potential relationship of risk score and drug sensitivity, the “oncoPredict” package was utilized to predict the drug sensitivity of PC patients. The results indicated that PC with low scores had higher sensitivity to cisplatin, epirubicin, fludarabine, irinotecan, KRAS (G12C) Inhibitor-12, oxaliplatin, and sorafenib (Figures 10A–G). On the other hand, PC with high scores exhibited higher sensitivity to trametinib and sapitinib (Figures 10H, I).

### 3.9 Experimental validation and single-cell analysis

**FIGURE 11:**
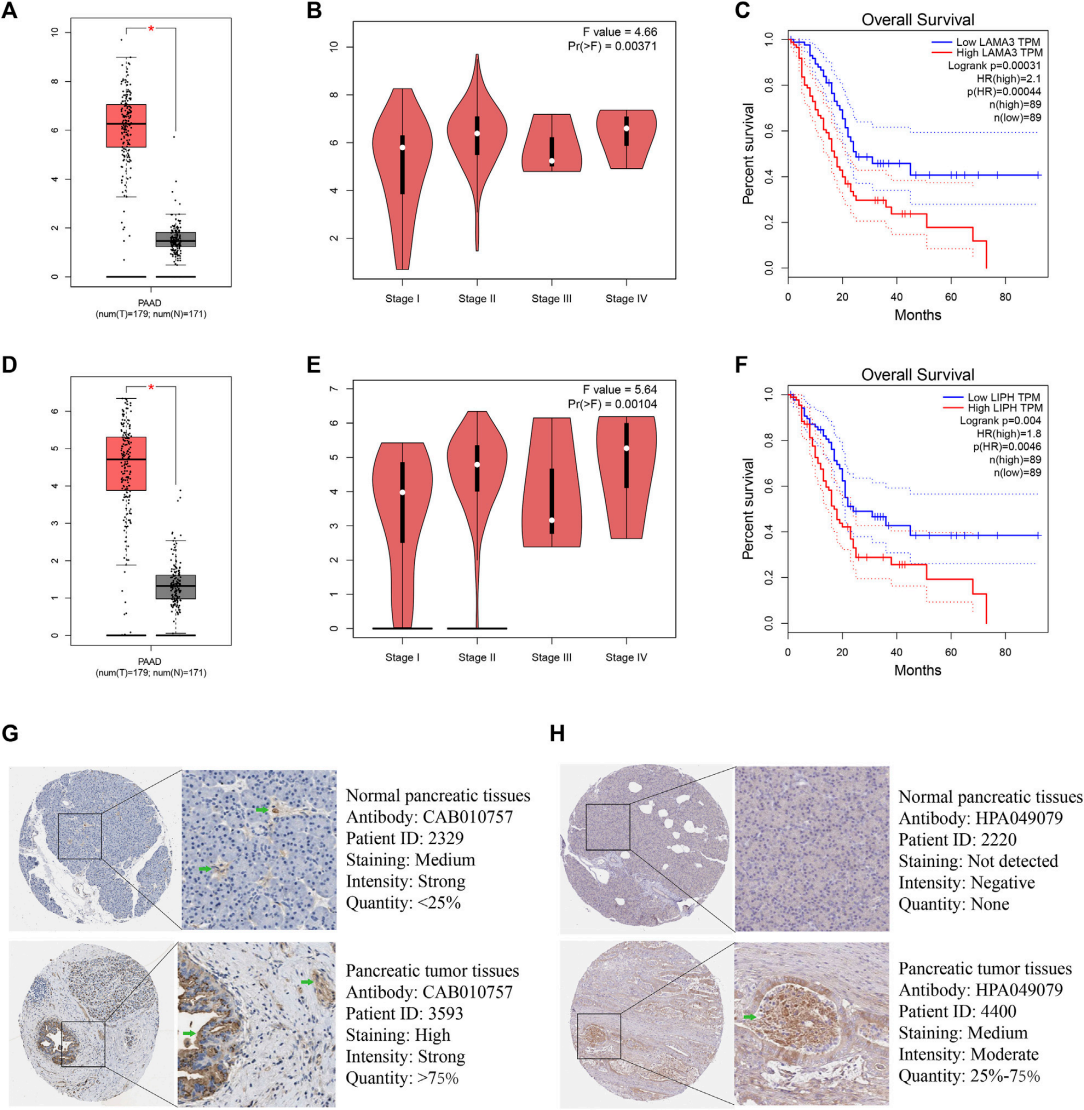
LAMA3 and LIPH. The expressed levels of LAMA3 in PC tumour tissue was remarkably higher within mRNA level **(A)**, and it was linked to TNM stage **(B)** and poor prognosis **(C)**. The expression of LIPH in PC tumour tissue was significantly higher within mRNA level **(D)**, and it was linked to TNM stage **(E)** and poor prognosis **(F)**. Immunohistochemical images indicated that the expression of LAMA3 **(G)** and LIPH **(H)** was significantly higher in pancreatic tumour tissues. (*p < 0.05).

**FIGURE 12:**
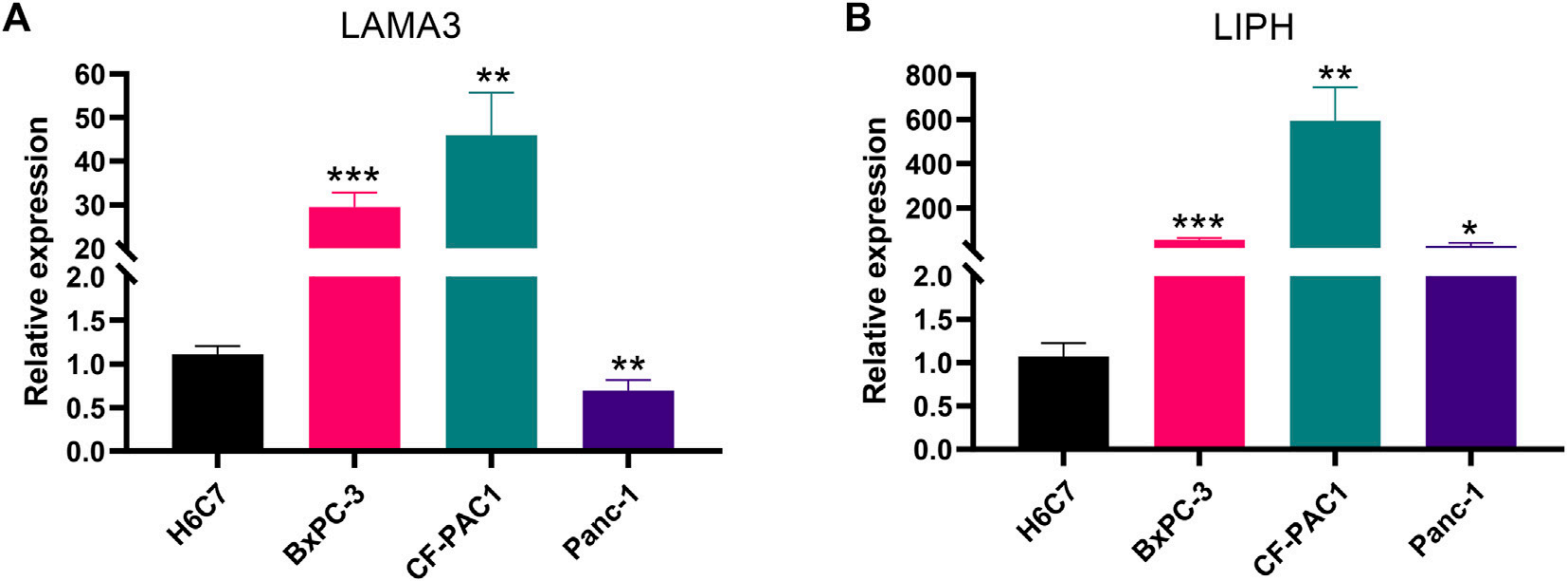
The validation of gene expression. LAMA3 **(A)** and LIPH **(B)** have higher expressed levels in PC cell lines than normal pancreatic cell lines. (*p < 0.05; **p < 0.01; ***p < 0.001).

**FIGURE 13:**
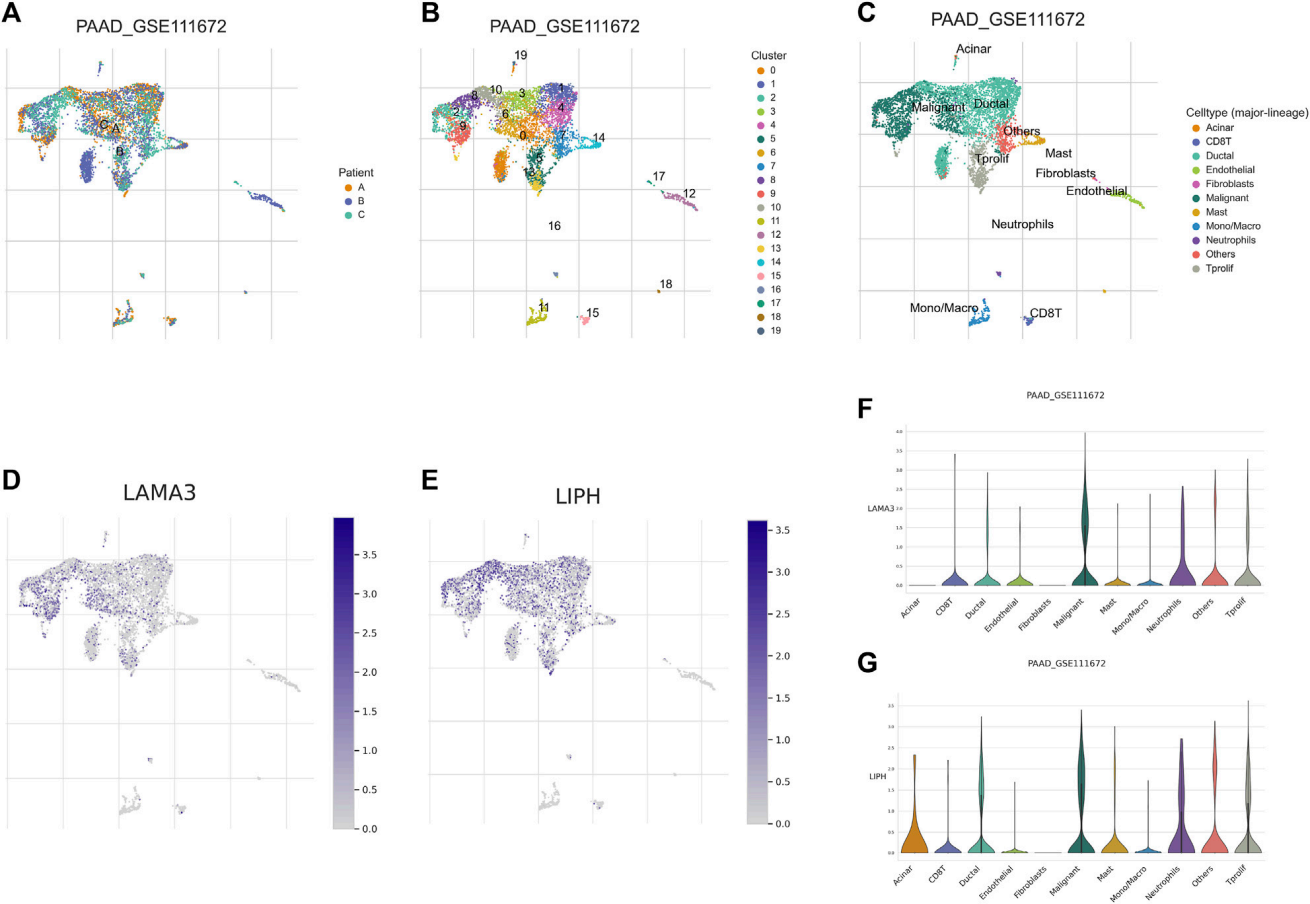
Single-cell analysis. **(A)** Annotation based on sample source. **(B)** Annotation based on cluster results. **(C)** Annotation based on the various cell subsets. **(D)** The distribution of LAMA3 expression within various cell subsets. **(E)** The distribution of LIPH expression within different cell subsets. **(F)** The relative expressed level of LAMA3 within various cell subsets. **(G)** The relative expressed level of LIPH within various cell subsets.

Utilizing GEPIA platform, the mRNA expression of LAMA3 and LIPH between pancreatic tumour and normal tissues was investigated. The expression of LAMA3 was markedly increased within pancreatic tumour tissues (Figure 11A) and was linked to the advanced stage and poor prognosis (Figures 11B, C). Similarly, LIPH also exhibited higher expression in pancreatic tumour tissues (Figure 11D) and was linked to the advanced stage and poor prognosis (Figures 11E, F). Furthermore, the immunohistochemistry images from HPA database showed that compared to normal pancreatic tissues, the protein expression of LAMA3 and LIPH was higher within PC tissues (Figures 11G, H). To further validate the reliability of our study, we performed qRT-PCR to confirm the expression of LAMA3 and LIPH. Similarly, LAMA3 and LIPH had higher expressed levels in PC cell lines than normal pancreatic cell lines (Figures 12A, B). Lastly, we performed single-cell analysis using the single-cell dataset GSE111672 (containing 3 samples and 6122 cells) to further uncover the cell subpopulations within the tumour microenvironment of PC. In addition to cancer cells, the tumour microenvironment of PC is also rich in monocytes/macrophages, endothelial cells, and fibroblasts (Figures 13A–C). LIPH and LAMA3 exhibited relatively higher expression in cancer cells and neutrophils (Figures 13D–G).

## 4 Discussion

PC, a highly lethal malignancy characterized by early metastasis and resistance to anticancer treatments, has become the seventh most common cause of cancer-related death globally. Despite the rapid development of diagnostic and therapeutic strategies for malignancies, patients with PC are frequently discovered at a late stage, and current treatments have little effect (Zhu et al., 2018). Therefore, there is significant clinical value in developing biomarkers for PC early diagnosis and risk assessment. Recently, the regulatory function of microbiome in cancer occurrence and development has been intensively studied, which can influence the occurrence, progression, metastasis as well as therapy response in various tumours. In this study, the important role of microbiome in the outcome, tumour microenvironment heterogeneity, and treatment response for PC patients was investigated by integrating microbiome and transcriptome data, and first constructed the microbiome-related subtypes and signature in PC.

We constructed the intratumor microbiome-derived subtypes by consensus cluster analysis. Survival analysis results suggested that Mcluster A had a remarkably better outcome compared with Mcluster B. What’s more, compared with Mcluster B, the proportion of T1-2 stages, N0 stages, M0 stages, Stage I, and pathological grade G1 in Mcluster A was higher. Next, we analyzed the abundance differences of 26 genera in the two subtypes, and found that Azohydromonas, Derxia, Holospora, Hylemonella, Paucibacter, Silanimonas, Starkeya, and Xanthobacter have significantly higher abundance in Mcluster A, and Alpharetrovirus, Indibacter, Riemerella, and Ruegeria have significantly higher abundance in Mcluster B. It has been reported that Alpharetrovirus can achieve almost complete elimination of leukemia cells by enhancing the toxicity of NK cells to leukemia cells (Suerth et al., 2016). We speculated that the tumour cells of PC patients with Mcluster B were more malignant. In order to enhance the killing ability of NK cells to fight against tumour cells, the body upregulated the level of Alpharetrovirus. Based on the above findings, microbiome was tightly connected with PC patients’ outcomes.

Then, we delved into the molecular mechanisms underlying the differences in prognosis of patients with different subtypes of PC. GSEA revealed that signaling pathways associated with ion-gated channels in tumour cells in patients with Mcluster A were remarkably enriched, while the activity of immune response signaling pathways in patients with Mcluster B were significantly enhanced. Ion-gated channels are responsible for tumour cell proliferation and are key factors in PC progression and invasion (Yee et al., 2012; Liu et al., 2018), and are also a key therapeutic target for PC (Yee, 2016). The microbiome with high abundance in Mcluster A patients may influence the prognosis of PC by regulating ion-gated channels. What’s more, previous studies have revealed that microbiome can participate in the immune response, which resulted in the prognostic change in patients with PC. Therefore, microbiome can influence the progression as well as outcome of PC through regulating ion-gated channels or immune response pathways. We further analyzed the relationship between intratumor microbiome-derived subtypes and tumour microenvironment heterogeneity, and found that Mcluster B had higher stromal and ESTIMATE scores. Neutrophil, Treg, CD8^+^ T cell, macrophages M1 and M2, cancer associated fibroblasts, myeloid dendritic cell, as well as activated mast cell had remarkably higher infiltrated levels within Mcluster B. CD8^+^ T cells can recognize and eliminate tumor cells through multiple mechanisms (Borst et al., 2018; Terrén et al., 2019; Philip and Schietinger, 2022). Studies indicated that the higher infiltrated levels of CD8^+^ T cell were linked to significantly longer survival time of PC patients (Carstens et al., 2017). Therefore, intratumor microbiome are likely to participate in shaping the tumour immune microenvironment, thereby affecting the immune response of tumor cells. The response of different intratumor microbiome-derived subtypes to immunotherapy was evaluated in this study. We found that the use of a CTLA-4 blocker was found to be more likely to be beneficial for PC patients in Mcluster B. Therefore, the study of microbiome in the tumour microenvironment of PC can help provide new strategies for the selection of immunotherapy for patients with PC.

For accurately predicting the prognosis for every PC patient, we utilized microbiome-related DEGs to construct and validate a prognostic signature. KM curves indicated that the survival time of PC patients with high-risk scores was remarkably lower, and ROC curves also indicated that the signature had a good predictive performance. What’s more, we explored the differences in the abundance of microbiome between the high and low-risk categories, and found that Riemerella had a significantly higher abundance in PC with high-risk scores and was linked to the poor outcome of PC. Riemerella is a Gram-negative rod-shaped bacterium that can cause acute infectious disease as well as an inflammatory response (Afrin et al., 2018; Li et al., 2023). However, Riemerella has not been reported in PC. Our study provides a new therapeutic target for PC. Correlation analysis indicated that the abundance of Riemerella was significantly linked positively to the expression of LIPH and LAMA3. LIPH is a new member of the triglyceride lipase family located on human chromosomes. The protein encoded by LIPH can hydrolyze triglycerides and phospholipids to produce fatty acids, which can then promote intestinal absorption or serve as an energy source or energy reserve (Jin et al., 2002). Studies indicated that LIPH had a higher expressed level in breast tumor tissue, and it affected the distant metastasis of breast cancer by regulating CAPN2 and paxillin (Seki et al., 2014; Zhang et al., 2020). According to the findings of our investigation, LIPH can be exploited as a possible therapeutic target for PC because it was found to be increased expression in PC tissues and to be related to the disease’s progression and bad prognosis. The LAMA3 gene can encode the α3 chain of laminin-5, which is an important cell membrane component and regulates cell adhesion and migration (Zhang et al., 2018; Xu et al., 2019). Studies indicated that LAMA3 is a promising target for cancer therapy since it may accelerate the growth and invasion of tumour cells (Xu et al., 2019; Shu et al., 2023). This agrees with our research suggesting that LAMA3 might be a useful treatment target for PC.

Drug-assisted therapy is one of the main ways to treat PC and can help improve the prognosis of patients. For example, modified FOLFIRINOX (containing oxaliplatin, irinotecan, leucovorin, and fluorouracil) and gemcitabine, as first-line chemotherapy regimens for PC, could result in 5-year disease-free survival rates of 26% and 19%, respectively, for patients with PC after surgery (Conroy et al., 2022). But the intricate PC tumour microenvironment frequently promotes the development of treatment resistance, which ultimately results in the failure of medication therapy. To improve treatment efficacy and prognosis, it is crucial to determine the medications to which each patient is sensitive. Our research assessed the relationship between intratumor microbiome-related subtypes and drug sensitivity. PC patients in Mcluster B or high-risk group had higher sensitivity to sapitinib, but PC patients in Mcluster A or low-risk group had higher sensitivity to cisplatin, irinotecan, oxaliplatin, sorafenib, and epirubicin. These findings provide a basis for individualized treatment of PC patients and are of great significance for improving the efficiency of drug treatment. The anti-tumor medication sapitinib has dual anti-tumor actions and can act on tumour blood vessels and tumour cells simultaneously (Gao et al., 2020; Attwa et al., 2023). Whether its curative effect on PC will be affected by intratumor microbiome and the specific mechanism still needs further basic research to explore.

However, our research has a few limitations that should be acknowledged. Firstly, this study belonged to retrospective research and was performed mainly based on data from public databases. Therefore, the prediction capability of our prognostic model should be validated in the prospective clinical research with large samples. Secondly, further investigation of molecular mechanism is required for exploring the function of intratumor microbiome in the occurrence and development of PC.

## 5 Conclusion

In the present study, we first constructed intratumor microbiome-derived subtypes in PC, and clarified the crucial role of microbiome in the outcome, tumor microenvironment shaping, and immunotherapy response for PC through multi-omics analysis, providing the novel microbiome-related targets for the treatment of PC. Meanwhile, we also built a prognostic signature utilizing intratumor microbiome-related genes to predict PC patients’ outcomes. In conclusion, this study can provide a novel insight for the prognosis prediction and treatment decision-making of PC.

## Data Availability

The datasets presented in this study can be found in online repositories. The names of the repository/repositories and accession number(s) can be found in the article/Supplementary Material.

## Supplementary material

SUPPLEMENTARY FIGURE S1 The relationship among the 26 prognostic-related genera.

SUPPLEMENTARY FIGURE S2 The cumulative distribution function (CDF) curve.

SUPPLEMENTARY FIGURE S3 The alteration of the area under CDF curve.

SUPPLEMENTARY FIGURE S4 Univariable Cox regression found 21 genes linked to the prognosis.

SUPPLEMENTARY TABLE S1 The abundance of 1406 genera in each pancreatic cancer sample.

SUPPLEMENTARY TABLE S2 44 prognostic genera were identified using the Kaplan–Meier method.
