# Supplementary figures and images for: Integrated multi-omics identified the novel intratumor microbiome-derived subtypes and signature to predict the outcome, tumor microenvironment heterogeneity, and immunotherapy response for pancreatic cancer patients
Source: Front Pharmacol. 2023 Sep 7;14:1244752. doi: 10.3389/fphar.2023.1244752 (PMC10512958; doi:10.3389/fphar.2023.1244752)

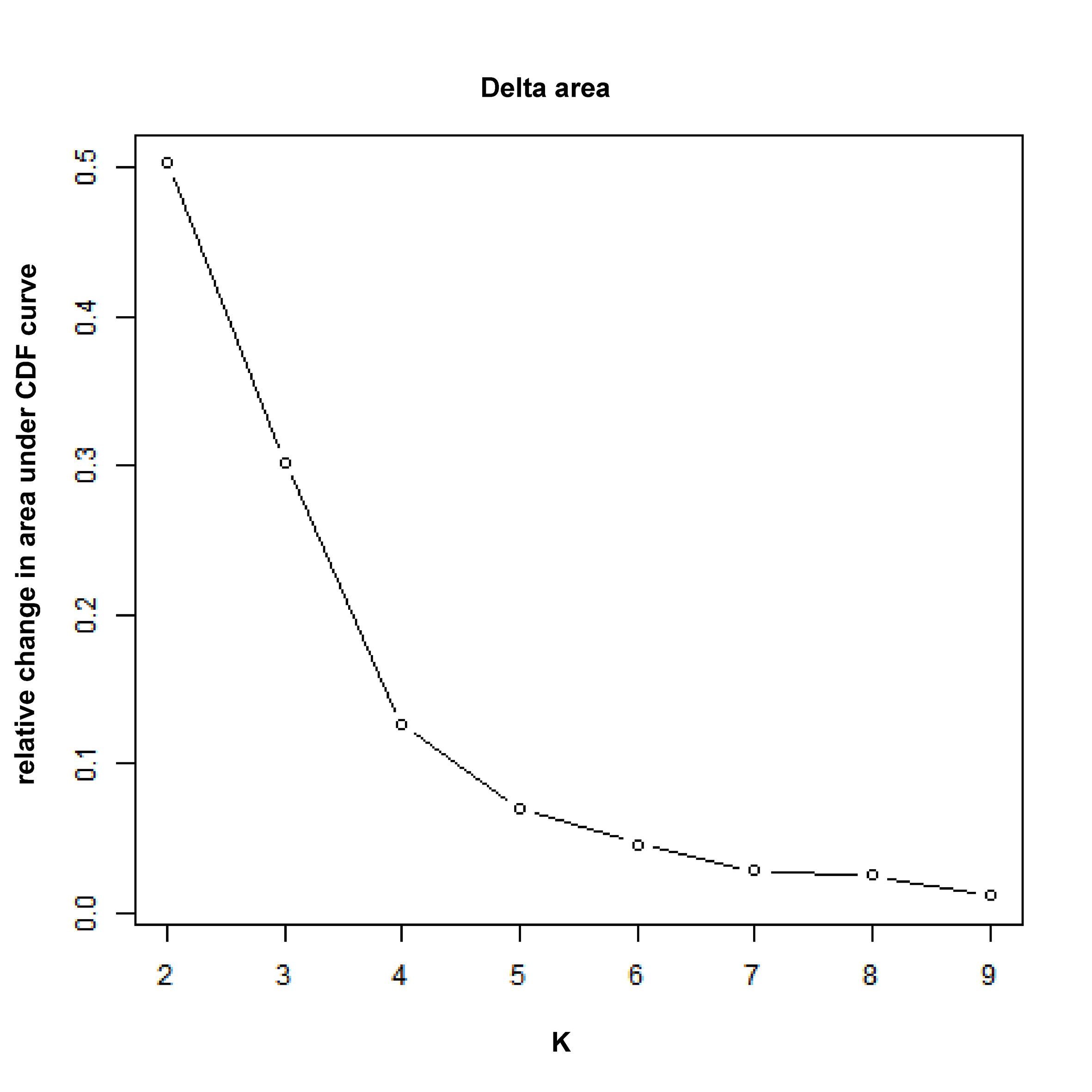

Supplement: Supplementary file 2 [file Image3.JPEG]

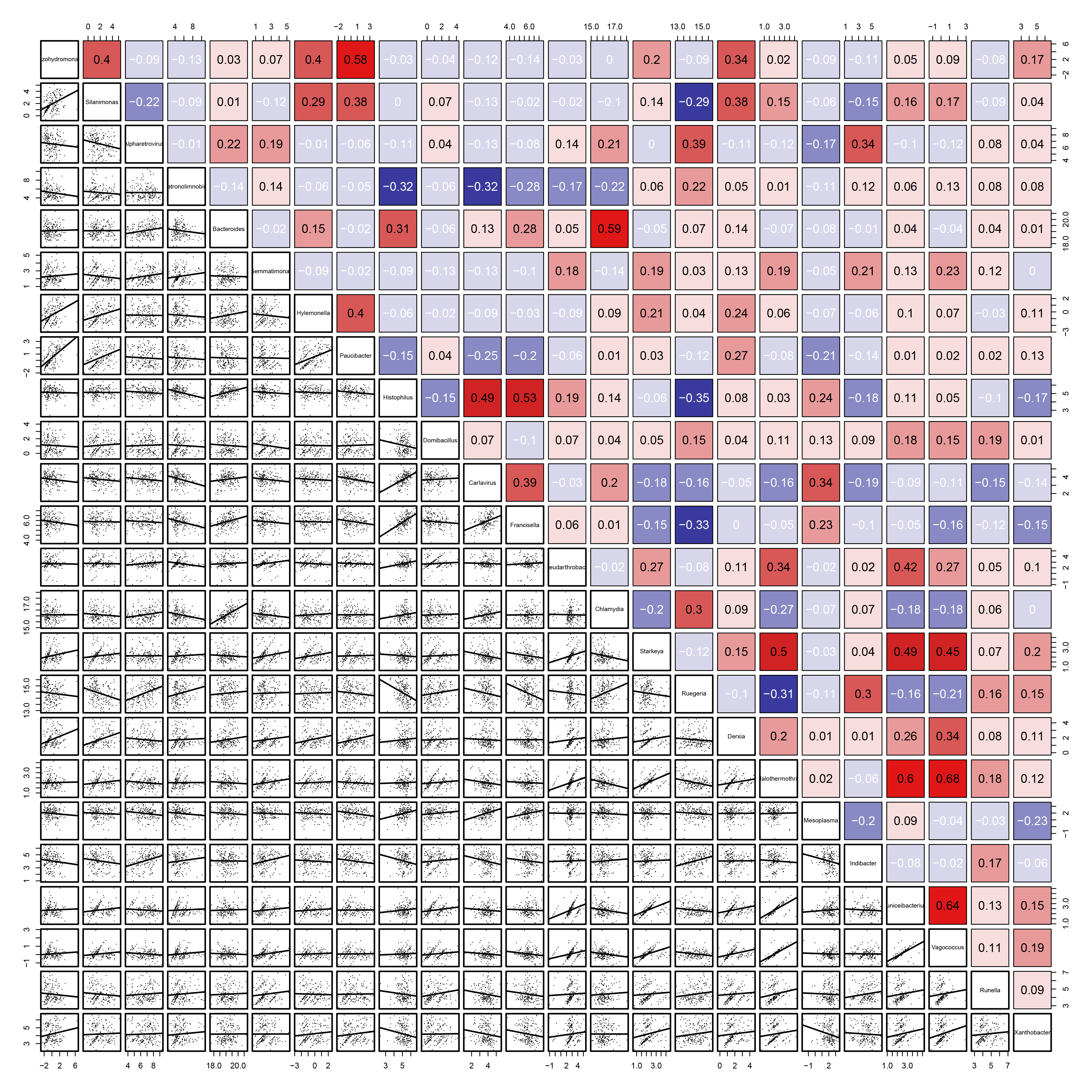

Supplement: Supplementary file 3 [file Image1.JPEG]

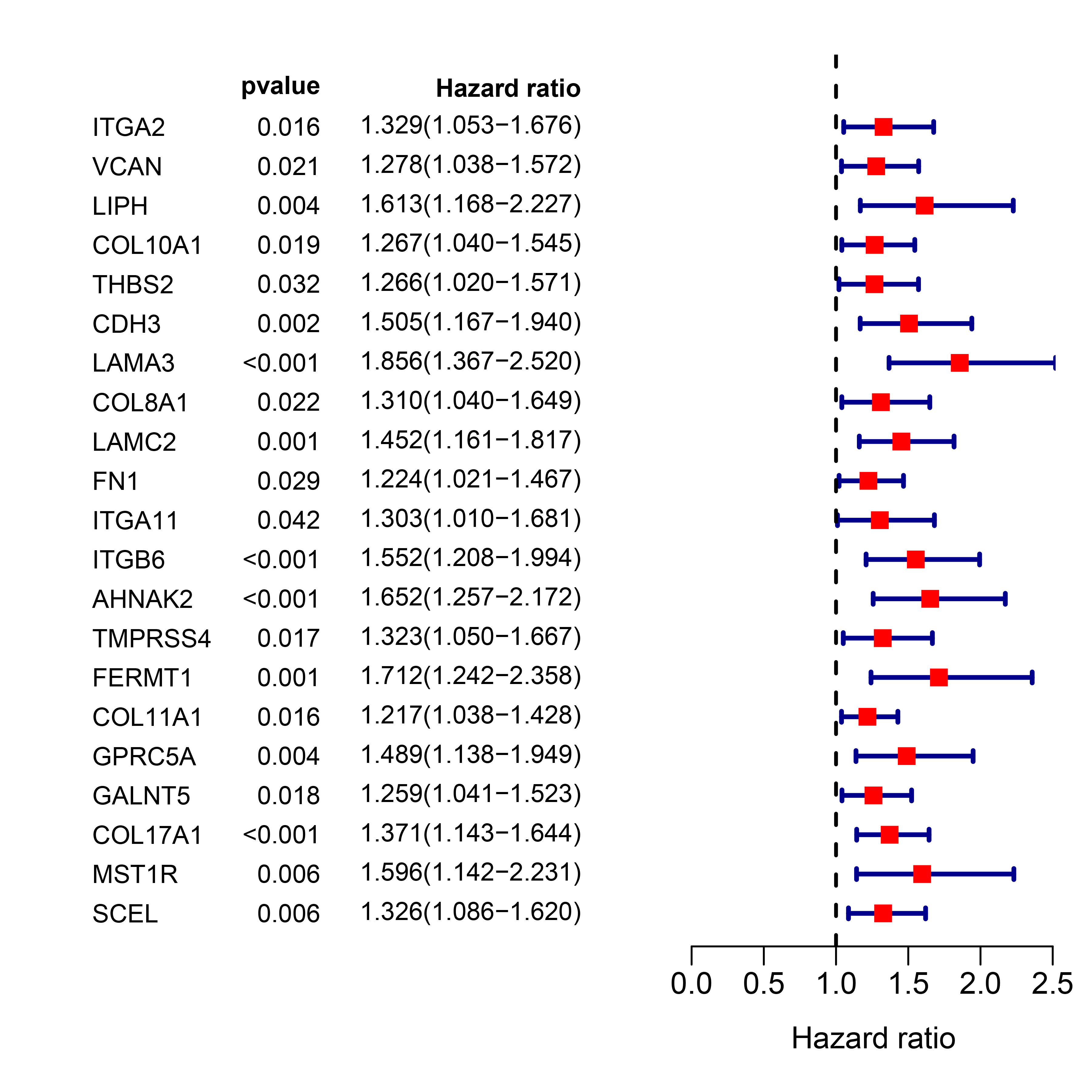

Supplement: Supplementary file 4 [file Image4.JPEG]

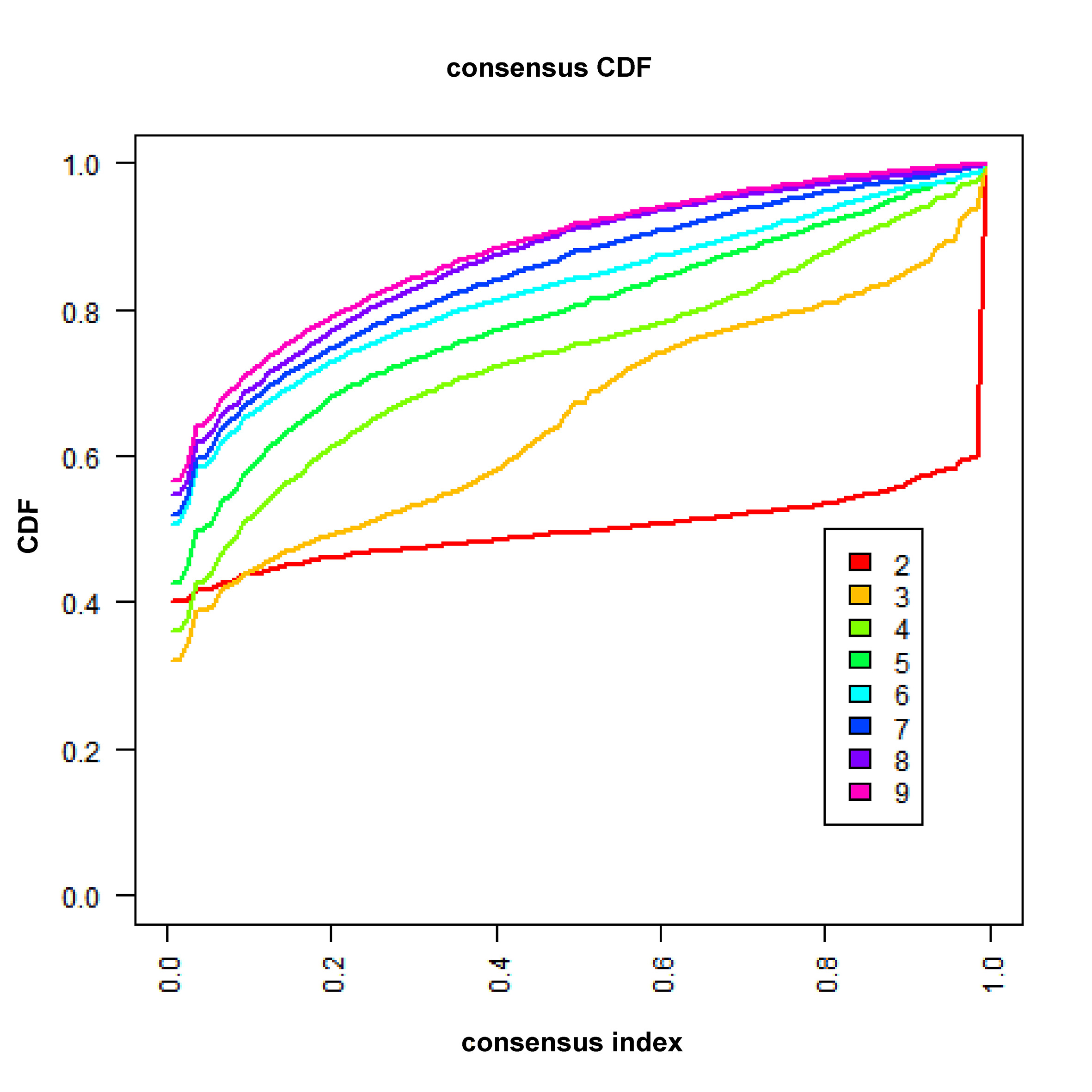

Supplement: Supplementary file 5 [file Image2.JPEG]
